# Association of Sickle Cell Trait With Incidence of Coronary Heart Disease Among African American Individuals

**DOI:** 10.1001/jamanetworkopen.2020.30435

**Published:** 2021-01-05

**Authors:** Hyacinth I. Hyacinth, Nora Franceschini, Samantha R. Seals, Marguerite R. Irvin, Ninad Chaudhary, Rakhi P. Naik, Alvaro Alonso, Cara L. Carty, Gregory L. Burke, Neil A. Zakai, Cheryl A. Winkler, Victor A. David, Jeffrey B. Kopp, Suzanne E. Judd, Robert J. Adams, Beatrice E. Gee, W. T. Longstreth, Leonard Egede, Daniel T. Lackland, Charles S. Greenberg, Herman Taylor, JoAnn E. Manson, Nigel S. Key, Vimal K. Derebail, Abhijit V. Kshirsagar, Aaron R. Folsom, Suma H. Konety, Virginia Howard, Matthew Allison, James G. Wilson, Adolfo Correa, Degui Zhi, Donna K. Arnett, George Howard, Alexander P. Reiner, Mary Cushman, Monika M. Safford

**Affiliations:** 1 Aflac Cancer and Blood Disorder Center, Department of Pediatrics, Emory University School of Medicine, Atlanta, Georgia; 2 Department of Epidemiology, University of North Carolina, Chapel Hill; 3 Department of Mathematics and Statistics, University of West Florida, Pensacola; 4 School of Public Health, University of Alabama at Birmingham, Birmingham; 5 Division of Hematology, Department of Medicine, The Johns Hopkins University, Baltimore, Maryland; 6 Department of Epidemiology, Rollins School of Public Health, Emory University, Atlanta, Georgia; 7 Fred Hutchinson Cancer Research Center, Seattle, Washington; 8 Department of Public Health Sciences, Wake Forest University, Winston-Salem, North Carolina; 9 Department of Medicine and Pathology and Laboratory Medicine, University of Vermont, Burlington; 10 Basic Science Laboratory, National Cancer Institute and Frederick National Laboratory, Leidos Biomedical Research, Frederick, Maryland; 11 National Institute of Diabetes and Digestive and Kidney Diseases, National Institutes of Health, Bethesda, Maryland; 12 Stroke Center, Department of Neurology, Medical University of South Carolina, Charleston; 13 Department of Neurology, University of Washington, Seattle; 14 Department of Epidemiology, University of Washington, Seattle; 15 Division of General Internal Medicine, Medical College of Wisconsin, Milwaukee; 16 Division of Hematology-Oncology, Medical University of South Carolina, Charleston; 17 Cardiovascular Research Institute, Morehouse School of Medicine, Atlanta, Georgia; 18 Department of Medicine, Brigham and Women’s Hospital, Harvard Medical School, Boston, Massachusetts; 19 Division of Hematology/Oncology, University of North Carolina at Chapel Hill, Chapel Hill; 20 University of North Carolina Kidney Center, University of North Carolina at Chapel Hill, Chapel Hill; 21 Division of Epidemiology and Community Health, University of Minnesota School of Public Health, Minneapolis; 22 Division of Cardiology, University of Minnesota Medical Center, Minneapolis; 23 Department of Epidemiology, School of Public Health, University of Alabama at Birmingham, Birmingham; 24 Department of Family Medicine and Public Health, University of California San Diego, San Diego; 25 Department of Physiology and Biophysics, University of Mississippi Medical Center, Jackson; 26 Jackson Heart Study, University of Mississippi Medical Center, Jackson; 27 Department of Biostatistics, School of Public Health, University of Alabama at Birmingham, Birmingham; 28 College of Public Health, University of Kentucky, Lexington; 29 Department of Biostatistics, School of Public Health, University of Alabama at Birmingham, Birmingham; 30 University of Washington Fred Hutchinson Cancer Research Center, Seattle; 31 Division of General Internal Medicine, Weill Cornell Medicine, New York, New York

## Abstract

**Importance:**

The incidence of and mortality from coronary heart disease (CHD) are substantially higher among African American individuals compared with non-Hispanic White individuals, even after adjusting for traditional factors associated with CHD. The unexplained excess risk might be due to genetic factors related to African ancestry that are associated with a higher risk of CHD, such as the heterozygous state for the sickle cell variant or sickle cell trait (SCT).

**Objective:**

To evaluate whether there is an association between SCT and the incidence of myocardial infarction (MI) or composite CHD outcomes in African American individuals.

**Design, Setting, and Participants:**

This cohort study included 5 large, prospective, population-based cohorts of African American individuals in the Women’s Health Initiative (WHI) study, the Reasons for Geographic and Racial Differences in Stroke (REGARDS) study, the Multi-Ethnic Study of Atherosclerosis (MESA), the Jackson Heart Study (JHS), and the Atherosclerosis Risk in Communities (ARIC) study. The follow-up periods included in this study were 1993 and 1998 to 2014 for the WHI study, 2003 to 2014 for the REGARDS study, 2002 to 2016 for the MESA, 2002 to 2015 for the JHS, and 1987 to 2016 for the ARIC study. Data analysis began in October 2013 and was completed in October 2020.

**Exposures:**

Sickle cell trait status was evaluated by either direct genotyping or high-quality imputation of rs334 (the sickle cell variant). Participants with sickle cell disease and those with a history of CHD were excluded from the analyses.

**Main Outcomes and Measures:**

Incident MI, defined as adjudicated nonfatal or fatal MI, and incident CHD, defined as adjudicated nonfatal MI, fatal MI, coronary revascularization procedures, or death due to CHD. Cox proportional hazards regression models were used to estimate the hazard ratio for incident MI or CHD comparing SCT carriers with noncarriers. Models were adjusted for age, sex (except for the WHI study), study site or region of residence, hypertension status or systolic blood pressure, type 1 or 2 diabetes, serum high-density lipoprotein level, total cholesterol level, and global ancestry (estimated from principal components analysis).

**Results:**

A total of 23 197 African American men (29.8%) and women (70.2%) were included in the combined sample, of whom 1781 had SCT (7.7% prevalence). Mean (SD) ages at baseline were 61.2 (6.9) years in the WHI study (n = 5904), 64.0 (9.3) years in the REGARDS study (n = 10 714), 62.0 (10.0) years in the MESA (n = 1556), 50.3 (12.0) years in the JHS (n = 2175), and 53.2 (5.8) years in the ARIC study (n = 2848). There were no significant differences in the distribution of traditional factors associated with cardiovascular disease by SCT status within cohorts. A combined total of 1034 participants (76 with SCT) had incident MI, and 1714 (137 with SCT) had the composite CHD outcome. The meta-analyzed crude incidence rate of MI did not differ by SCT status and was 3.8 per 1000 person-years (95% CI, 3.3-4.5 per 1000 person-years) among those with SCT and 3.6 per 1000 person-years (95% CI, 2.7-5.1 per 1000 person-years) among those without SCT. For the composite CHD outcome, these rates were 7.3 per 1000 person-years (95% CI, 5.5-9.7 per 1000 person-years) among those with SCT and 6.0 per 1000 person-years (95% CI, 4.9-7.4 per 1000 person-years) among those without SCT. Meta-analysis of the 5 study results showed that SCT status was not significantly associated with MI (hazard ratio, 1.03; 95% CI, 0.81-1.32) or the composite CHD outcome (hazard ratio, 1.16; 95% CI, 0.92-1.47).

**Conclusions and Relevance:**

In this cohort study, there was not an association between SCT and increased risk of MI or CHD in African American individuals. These disorders may not be associated with sickle cell trait–related sudden death in this population.

## Key Points

**Question:**

Is sickle cell trait associated with increased risk of myocardial infarction and coronary heart disease among African American individuals?

**Findings:**

In this cohort study of 23 197 African American individuals in 5 cardiovascular epidemiologic studies, sickle cell trait was not associated with increased risk of myocardial infarction or coronary heart disease among African American individuals.

**Meaning:**

In this study, sickle cell trait was not associated with increased risk of fatal and nonfatal myocardial infarction or coronary heart disease, suggesting that these disorders may not be associated with sickle cell trait–related sudden death.

## Teaser

This cohort study evaluates whether African American individuals with sickle cell trait have a higher incidence of myocardial infarction or composite coronary heart disease outcomes compared with those without sickle cell trait.

## Introduction

The burden of cardiovascular disease (CVD) in general and coronary heart disease (CHD) in particular is disproportionately high among African American individuals,^1,2^ resulting in a substantial health disparity.^3,4,5^ Approximately 60% of this disparity is associated with the excess burden of classical factors associated with CVD among African American individuals compared with non-Hispanic White individuals.^3,4,6,7,8,9,10^ The associations of hypertension with CHD^11^ and of the apolipoprotein L1 (*APOL1* [RefSeq ID NG_023228]) risk gene variant (ie, G1 [rs73885319 and rs60910145] and/or G2 [rs71785313]) with excess burden of hypertensive kidney disease among African American individuals^12,13,14^ have raised the question of a possible association between genetic variation and some of the observed excess and unexplained burden of CHD among African American individuals compared with non-Hispanic White individuals. Ito et al^15^ reported that possession of 2 *APOL1* risk alleles was associated with higher overall risk for CVD and with early age at onset among African American individuals. Although there have been inconsistent findings for the association between *APOL1* genotypes and CVD,^16^ there is a rationale that genetic variation is possibly associated with the observed disparities in the incidence and prevalence of CVD in general and CHD in particular among African American individuals. Several studies have reported that in addition to the *APOL1* gene variant, heterozygosity for the sickle cell variant (sickle cell trait [SCT]) was associated with the incidence and progression of chronic kidney disease (CKD) and albuminuria,^17,18,19^ a factor associated with CVD.^20,21^

Sickle cell trait results from the genetic inheritance of 1 normal and 1 sickle β-globin gene (ie, the heterozygous state for the sickle β-globin gene). Approximately 8% of African American individuals and 20% to 35% of African individuals are SCT carriers. The variant is attributable to a functional single-nucleotide variant involving the substitution of GTG (valine) for GAG (glutamic acid) at the sixth amino acid position in the gene encoding β-globin. Long considered a silent or benign carrier state, some studies have suggested a possible association between SCT and clinical complications.^22,23^ For instance, among African American individuals with end-stage kidney disease, the prevalence of SCT was higher compared with that among those without the disease.^19^ Similarly, SCT is associated with a higher risk for CKD and albuminuria,^17,24^ kidney medullary carcinoma,^25,26^ venous thromboembolism in various clinical contexts,^27,28,29^ and pulmonary embolism.^30^ Furthermore, individuals with SCT may have a prothrombotic state characterized by higher serum levels of C-reactive protein,^31^ F2.1 fragments, thrombin-antithrombin complex, and d-dimer^32^; these biomarkers have been associated with an increased risk of CHD. Thus, given the association between CKD and CVD^20,33,34^ and between SCT and CKD or end-stage kidney disease,^17,18,19,24^ we hypothesized that among African American individuals, the presence of SCT would be associated with a higher incidence of myocardial infarction (MI) and/or CHD. We also examined whether African American individuals with SCT, compared with those without SCT, had a higher risk of MI or CHD after adjustment for traditional factors associated with CHD.

## Methods

### Study Sample

This cohort study included 5 large population-based cohort studies: the Women’s Health Initiative (WHI) study,^35^ the Reasons for Geographic and Racial Differences in Stroke (REGARDS) study,^36^ the Multi-Ethnic Study of Atherosclerosis (MESA),^37^ the Jackson Heart Study (JHS),^38,39^ and the Atherosclerosis Risk in Communities (ARIC) study.^40,41^ The study sample consisted of participants in these studies who self-identified as African American. The design and methods of each study have been previously described^35,37,38,39,40,41,42^ and are summarized in the eMethods in the Supplement. Only participants without evidence of CHD at baseline either by medical history or electrocardiography were included in the analyses. The follow-up periods for each of the cohorts included in this study were 1993 and 1998 to 2014 for the WHI study, 2003 to 2014 for the REGARDS study, 2002 to 2016 for the MESA, 2002 to 2015 for the JHS, and 1987 to 2016 for the ARIC study. The sample from the JHS excluded participants who were also in the ARIC study cohort. All participants included in these studies provided prior written informed consent for genetic studies, and institutional review board approval was obtained by each cohort at each participating institution. This study followed the Strengthening the Reporting of Observational Studies in Epidemiology (STROBE) reporting guideline.

### Exposure Assessment

The primary exposure for this study was presence of the rs334 single-nucleotide variant, which causes the variant leading to the formation of hemoglobin S or sickle hemoglobin and the amino acid substitution (*HBB* p.Glu7Val). Assessment of SCT status was done by direct custom genotyping (n = 19 814), exome sequencing (n = 1979), or imputation into the remaining sample of African American individuals with genome-wide single-nucleotide variant genotyping (Affymetrix 6.0; Thermo Fisher Scientific) (n = 1404) (eMethods in the Supplement). Participants who were homozygous for rs334 (ie, had sickle cell anemia) or were compound heterozygotes (ie, inherited 1 sickle cell gene and 1 hemoglobinopathy gene), including 1 participant in the ARIC study, 3 in the JHS, and 2 in the WHI study, were excluded from the analyses. After further excluding participants with missing genotype, CHD outcomes, or relevant covariate information and after ensuring no overlap of participants (between the JHS and the ARIC study), a combined total of 23 197 participants were included in this study (5904 from the WHI study, 10 714 from the REGARDS study, 1556 from the MESA, 2175 from the JHS, and 2848 from the ARIC study).

### Covariate Assessment

Each study (cohort) administered questionnaires, a physical examination, and physiological assessment at baseline. Data collected included participants’ sociodemographic information, health behaviors, and medical and medication history. Physical examination included blood pressure, height, and weight measurements. Physiological assessment included testing of blood samples for fasting blood glucose and lipid profiles.

Factors associated with CHD included hypertension, type 1 or 2 diabetes, cigarette smoking, and serum total cholesterol and high-density lipoprotein levels. Hypertension was defined as a baseline systolic blood pressure of 140 mm Hg or more, a diastolic blood pressure of 90 mm Hg or more, or self-reported use of antihypertensive medication. Diabetes was defined as a baseline fasting glucose level of 126 mg/dL or more (to convert to millimoles per liter, multiply by 0.0555), a nonfasting glucose level of 200 mg/dL or more, self-reported physician diagnosis of diabetes, or self-reported use of oral hypoglycemic medication or insulin. Cigarette smoking status was defined as self-report of being a current smoker or not except in the WHI and ARIC studies, in which smoking status was based on history of smoking (ie, ever smokers vs never smokers). Serum lipid levels as a covariate was defined using total cholesterol levels, high-density lipoprotein cholesterol levels, or both.

Other covariates included annual household income, educational level, C-reactive protein concentration, use of statins at baseline, use of aspirin at baseline, and baseline estimated glomerular filtration rate. Population substructure was assessed through estimation of global ancestry using principal components analysis derived from genome-wide genotyping data.^43^ Adjustment for population substructure was done using eigenvalues derived from the principal components analysis.

### Assessment of CHD Outcomes

Adjudicated CHD outcomes were obtained during follow-up in each cohort and, for the purpose of our study, were grouped into 2 outcomes. The first was MI, including fatal and nonfatal MI events. The second was a composite CHD outcome including nonfatal and fatal MI, other types of fatal CHD events, and coronary revascularization procedures. The clinical and laboratory criteria for assessing and adjudicating MI and CHD in each cohort are published elsewhere^35,37,41,42,44^ and summarized in the eMethods in the Supplement. All 5 cohorts used similar criteria for MI and CHD adjudication.

### Statistical Analysis

Data analysis began in October 2013 and was completed in October 2020. We tabulated baseline demographic characteristics, factors associated with CVD, and follow-up data stratified by SCT status and reported them as either proportions or means with SDs. The crude incidence of MI or CHD was estimated per 1000 person-years and then meta-analyzed using inverse variance weighting a priori assuming a random-effects model owing to the small number of cohorts (5) and potential heterogeneity among study cohorts because of differences in sampling frame and some covariate ascertainment. The association of SCT carrier status with incident MI or CHD was evaluated using Cox proportional hazards regression models and expressed as hazard ratios. The first set of analyses investigated the association between incident MI and SCT status. The second set of analyses investigated the association between incident composite CHD outcome and SCT status.

We performed a stepwise analysis with 4 levels of baseline covariate adjustment, resulting in 4 analytical models for each end point and for each cohort. In model 1, we adjusted for age, sex, principal components of global genetic ancestry (derived from the genome-wide array genotyping data), and region of residence for the REGARDS study (stroke belt [the 9 Southeastern states with the highest incidence of and mortality from stroke] vs nonstroke belt) and the ARIC study (Forsyth County vs Jackson) only because region was a sampling variable in both cohorts. In model 2, we also adjusted for the components of the Framingham CHD Risk Score (hypertension, diabetes, cigarette smoking status, and total cholesterol level, high-density lipoprotein cholesterol level, or both)^45,46^ in addition to the covariates in model 1. In model 3, we adjusted for all the covariates in model 2, in addition to adjusting for income, educational level, serum C-reactive protein level, self-reported baseline statin use, and self-reported baseline aspirin use. In the WHI study, we also adjusted for clinical trial or observational study participation. In model 4, we added estimated glomerular filtration rate to all the covariates in model 3. The results from the Cox proportional hazards regression models (ie, from each of models 1, 2, 3, and 4) were each meta-analyzed using inverse variance–weighted meta-analysis assuming a random-effects model. We also present the results of the meta-analysis of the fully adjusted model (model 4) in forest plots. Statistical analysis was performed with Stata, version 12 (StataCorp LLC). All *P* values were from 2-sided tests and results were deemed statistically significant at *P* < .05.

## Results

### Baseline Characteristics

A total of 23 197 African American men (29.8%) and women (70.2%) were included in the combined sample. The mean (SD) ages for participants at baseline were 61.2 (6.9) years in the WHI study, 64.0 (9.3) years in the REGARDS study, 62.2 (10.0) years in the MESA, 50.3 (12.0) years in the JHS, and 53.2 (5.8) years in the ARIC study. Of the total 23 197 participants, 1781 had SCT; the prevalence of SCT in our study ranged from 6.4% to 9.1% across cohorts, with a mean of 7.7%, which is consistent with the reported range of population prevalence of SCT in the US.^17,47,48^ There were no differences in the distribution of traditional factors associated with CVD by SCT status within each cohort-based comparison using a univariate χ^2^ test. Table 1 provides a detailed within-cohort comparison of baseline characteristics between participants with and without SCT.^35,36,37,38,39,40,41^

**Table 1.** Baseline Characteristics of Participants by Study Cohort and SCT Status

Variable	Participants^a^									
	WHI study^35^ (N = 5904)		REGARDS study^36^ (N = 10 714)		MESA^37^ (N = 1556)		JHS^38,39^ (N = 2175)		ARIC study^40,41^ (N = 2848)	
	SCT	No SCT	SCT	No SCT	SCT	No SCT	SCT	No SCT	SCT	No SCT
Frequency of rs334	485/5904 (8.2)	5419/5904 (91.8)	802/10 714 (7.5)	9912/10 714 (92.5)	141/1556 (9.1)	1415/1556 (90.9)	171/2175 (7.9)	2004/2175 (92.1)	182/2848 (6.4)	2666/2848 (93.6)
Age, mean (SD), y	61.1 (7.1)	61.2 (6.9)	63.5 (9.4)	64.0 (9.3)	61.0 (10.0)	62.0 (10.0)	50.9 (12.4)	50.3 (12.0)	53.4 (5.8)	53.2 (5.8)
Male	0	0	287/802 (35.8)	3836/9912 (38.7)	64/141 (45.4)	651/1415 (46.0)	75/171 (43.9)	768/2004 (38.3)	72/182 (39.6)	1171/2848 (41.1)
Systolic blood pressure, mm Hg, mean (SD)	130.7 (16.8)	131.3 (17.0)	131.6 (17.7)	130.9 (17.4)	131.0 (20.0)	132.0 (22.0)	123.6 (16.1)	125.5 (16.7)	129.0 (21.0)	128 (20.0)
Type 1 or 2 diabetes	67/485 (13.8)	585/5419 (10.8)	241/802 (30.0)	2896/9912 (29.2)	32/141 (22.7)	241/1415 (17.0)	31/171 (18.1)	376/2004 (18.8)	30/182 (16.5)	487/2666 (18.3)
Hypertension	243/485 (50.1)	2796/5419 (51.6)	555/802 (69.2)	7090/9912 (71.5)	80/141 (56.7)	835/1415 (59.0)	80/171 (46.8)	989/2004 (49.4)	100/182 (55.0)	1454/2666 (54.5)
Current cigarette smoking	233/485 (48.0)	2807/5419 (51.8)	141/802 (17.5)	1739/9912 (17.6)	27/141 (19.1)	269/1415 (19.0)	26/171 (15.2)	294/2004 (14.7)	43/182 (23.6)	792/2666 (29.7)
Atrial fibrillation^b^	0	0	9/802 (1.1)	67/9912 (0.7)	0	0	1/171 (0.6)	5/2004 (0.3)	0	2/2666 (0.1)
LVH^c^	NA	NA	50/802 (6.2)	502/9912 (5.1)	NA	NA	10/171 (5.9)	80/2004 (4.0)	8/182 (4.4)	127/2666 (4.8)
History of CVD	0	0	131/802 (16.3)	1510/9912 (15.2)	0	0	15/171 (8.8)	177/2004 (8.8)	NA	NA
All incident MI events	23/485 (4.7)	307/5419 (5.7)	21/802 (2.6)	207/9912 (2.1)	9/141 (6.4)	80/1415 (5.7)	5/171 (2.9)	47/2004 (2.4)	18/182 (9.9)	317/2666 (11.9)
Composite CHD events	30/485 (6.2)	461/5419 (8.5)	42/802 (5.2)	439/9912 (4.4)	17/141 (12.1)	107/1415 (7.6)	7/171 (4.1)	70/2004 (3.5)	41/182 (22.5)	500/2666 (18.8)
History										
Aspirin use	NA	NA	282/802 (35.2)	3814/9912 (38.5)	43/141 (30.5)	431/1415 (30.5)	29/171 (17.0)	379/2004 (18.9)	45/182 (24.7)	748/2666 (28.1)
Statin use	36/485 (7.4)	354/5419 (6.5)	233/802 (29.1)	2868/9912 (28.9)	28/141 (19.9)	194/1415 (13.7)	9/171 (5.3)	186/2004 (9.3)	0	6/2666 (0.2)
Follow-up time, median, y	13.3	13.2	8.9	9.4	13.6	13.8	11.5	11.6	25.3	26.0
Total follow-up time, person-years	5924.8	66 331.2	14 913	192 004	1918	19 527	1681.3	20 542.6	3970	58 590
Age at event, mean (SD), y										
MI	73.3 (7.6)	75.8 (8.9)	71.0 (8.3)	71.0 (9.0)	69.0 (9.7)	70.0 (10.0)	62.1 (7.4)	61.1 (11.9)	70.3 (8.9)	68.6 (8.7)
Any CHD	72.1 (7.5)	74.5 (7.9)	71.7 (9.2)	71.0 (9.1)	68.0 (9.5)	70.0 (10.0)	60.3 (6.8)	63.0 (12.6)	69.5 (9.3)	68.3 (8.2)

Abbreviations: ARIC, Atherosclerosis Risk in Communities; CHD, coronary heart disease; CVD, cardiovascular disease; JHS, Jackson Heart Study; LVH, left ventricular hypertrophy; MESA, Multi-Ethnic Study of Atherosclerosis; MI, myocardial infarction; NA, not applicable; REGARDS, Reasons for Geographic and Racial Differences in Stroke; rs334, gene variant that causes sickle cell anemia; SCT, sickle cell trait; WHI, Women’s Health Initiative.

^a^ Data are presented as number/total number (percentage) of participants unless otherwise indicated.

^b^ Atrial fibrillation was determined by self-report or electrocardiographic evidence.

^c^ Left ventricular hypertrophy was determined by 12-lead electrocardiography.

### Association of SCT With MI or CHD

A combined total of 1034 participants (76 with SCT) had incident MI and 1714 (137 with SCT) had the composite CHD outcome. Within-cohort analysis and subsequent meta-analysis showed that the crude incidence rates for MI among participants with SCT (3.8 per 1000 person-years; 95% CI, 3.3-4.5 per 1000 person-years) compared with those without SCT (3.6 per 1000 person-years; 95% CI, 2.7-5.1 per 1000 person-years) were not significantly different (Table 2).^35,36,37,38,39,40,41^ Similarly, significant differences were not found in the crude incidence of the composite CHD event among participants with SCT (7.3 per 1000 person-years; 95% CI, 5.5-9.7 per 1000 person-years) compared with those without SCT (6.0 per 1000 person-years; 95% CI, 4.9-7.4 per 1000 person-years).

**Table 2.** Crude Incidence of Myocardial Infarction and CHD Outcomes by Study Cohort and SCT Status

Outcome	Crude incidence, per 1000 person-years (95% CI)											
	WHI study^35^		REGARDS study^36^		MESA^37^		JHS^38,39^		ARIC study^40,41^		Meta-analysis	
	SCT	No SCT	SCT	No SCT	SCT	No SCT	SCT	No SCT	SCT	No SCT	SCT	No SCT
Myocardial infarction	3.9 (2.6-5.8)	4.6 (4.1-5.1)	3.7 (3.0-4.5)	2.8 (2.6-3.0)	4.7 (2.3-8.6)	4.1 (3.3-5.1)	3.0 (1.1-6.6)	2.3 (1.7-3.0)	4.5 (2.7-7.2)	5.4 (4.8-6.0)	3.8 (3.3-4.5)	3.6 (2.7-5.1)
Any CHD event	5.1 (3.5-7.2)	6.9 (6.3-7.6)	7.7 (6.3-9.4)	6.3 (5.9-6.7)	8.8 (5.3-13.9)	5.5 (4.5-6.6)	4.2 (1.8-8.2)	3.4 (2.7-4.3)	10.7 (7.7-14.5)	8.7 (7.9-9.5)	7.3 (5.5-9.7)	6.0 (4.9-7.4)

Abbreviations: ARIC, Atherosclerosis Risk in Communities; CHD, coronary heart disease; JHS, Jackson Heart Study; MESA, Multi-Ethnic Study of Atherosclerosis; REGARDS, Reasons for Geographic and Racial Differences in Stroke; SCT, sickle cell trait; WHI, Women’s Health Initiative.

The results of the fully adjusted multivariable models that also accounted for baseline kidney function (model 4) for each cohort are presented in Table 3 and Table 4.^35,36,37,38,39,40,41^ Meta-analysis of results from model 4 for incident MI gave a hazard ratio of 1.03 (95% CI, 0.81-1.32), indicating no significant association between SCT and incidence of MI (Table 3 and Figure, A).^35,36,37,38,39,40,41^ Similarly, meta-analysis of results from model 4 for CHD showed that the hazard ratio for incident CHD was 1.16 (95% CI, 0.92-1.47) (Table 4 and Figure, B).^35,36,37,38,39,40,41^ A priori, we assumed a random-effects model and retained the results of this model notwithstanding the fact that our test of heterogeneity indicated no significant heterogeneity between studies (*I*^2^ = 0.0%; *P* = .54 for the MI meta-analysis [Figure, A]; and *I*^2^ = 26.2%; *P* = .25 for the CHD meta-analysis [Figure, B]). This approach was adopted because heterogeneity exists between studies and the reliability of a test of heterogeneity with 5 studies is not robust and thus not reliable.

**Table 3.** Hazard Ratios for Myocardial Infarction Among Individuals With SCT Compared With Those Without SCT by Study Cohort

Model^a^	WHI study^35^		REGARDS study^36^		MESA^37^		JHS^38,39^		ARIC study^40,41^		Meta-analysis	
	HR (95% CI)	*P* value	HR (95% CI)	*P* value	HR (95% CI)	*P* value	HR (95% CI)	*P* value	HR (95% CI)	*P* value	HR (95% CI)	*P* value
Model 1	0.94 (0.61-1.43)	.76	1.47 (0.83-2.61)	.18	1.26 (0.63-2.52)	.51	1.19 (0.55-2.67)	.66	0.82 (0.51-1.32)	.41	1.04 (0.82-1.33)	.71
Model 2	0.98 (0.64-1.50)	.93	1.48 (0.83-2.63)	.18	1.21 (0.60-2.42)	.59	1.37 (0.62-3.02)	.44	0.83 (0.52-1.34)	.46	1.07 (0.84-1.37)	.59
Model 3^b^	1.00 (0.65-1.54)	.99	1.40 (0.77-2.54)	.27	1.21 (0.61-2.43)	.59	1.49 (0.67-3.32)	.33	0.82 (0.51-1.32)	.41	1.07 (0.83-1.37)	.60
Model 4^b^	0.98 (0.64-1.51)	.92	1.27 (0.70-2.31)	.43	1.28 (0.64-2.56)	.49	1.48 (0.67-3.32)	.33	0.77 (0.48-1.24)	.28	1.03 (0.81-1.32)	.80

Abbreviations: ARIC, Atherosclerosis Risk in Communities; CHD, coronary heart disease; HR, hazard ratio; JHS, Jackson Heart Study; MESA, Multi-Ethnic Study of Atherosclerosis; REGARDS, Reasons for Geographic and Racial Differences in Stroke; SCT, sickle cell trait; WHI, Women’s Health Initiative.

^a^ Model 1 adjusted for age, sex, study region (REGARDS and ARIC studies only), and principal components analysis of global genetic ancestry; model 2 adjusted for components of model 1 and components of the Framingham CHD risk score (hypertension [defined as diagnosed or current use of antihypertensive medication] or a high systolic blood pressure), diabetes, cigarette smoking (current vs noncurrent except in the WHI and ARIC studies, in which the data were ever smokers vs never smokers), and serum total cholesterol and/or high-density lipoprotein cholesterol levels; model 3 adjusted for components of model 2 and income, educational level, serum high-sensitivity C-reactive protein level, and history of statin and aspirin use (WHI analysis also adjusted for trial group among WHI participants); and model 4 adjusted for components of model 3 and glomerular filtration rate, estimated from the CKD-Epi equation.

^b^ For models 3 and 4 in the WHI study, the total sample size was 5744, with 473 individuals with SCT.

**Table 4.** Hazard Ratios for Combined CHD Outcomes Among Individuals With SCT Compared With Those Without SCT by Study Cohort

Model^a^	WHI study^35^		REGARDS study^36^		MESA^37^		JHS^38,39^		ARIC study^40,41^		Meta-analysis	
	HR (95% CI)	*P* value	HR (95% CI)	*P* value	HR (95% CI)	*P* value	HR (95% CI)	*P* value	HR (95% CI)	*P* value	HR (95% CI)	*P* value
Model 1	0.81 (0.56-1.17)	.26	1.35 (0.91-1.99)	.13	1.87 (1.11-3.12)	.01	1.03 (0.24-4.40)	.97	1.20 (0.87-1.65)	.27	1.20 (0.91-1.54)	.12
Model 2	0.83 (0.57-1.22)	.35	1.39 (0.94-2.05)	.10	1.77 (1.06-2.97)	.03	1.00 (0.23-4.37)	.99	1.20 (0.87-1.66)	.26	1.21 (0.93-1.56)	.15
Model 3^b^	0.86 (0.59-1.25)	.43	1.34 (0.90-2.00)	.15	1.68 (1.00-2.83)	.05	1.15 (0.26-5.15)	.85	1.20 (0.87-1.65)	.27	1.19 (0.95-1.48)	.13
Model 4^b^	0.85 (0.58-1.24)	.39	1.26 (0.84-1.87)	.27	1.80 (1.06-3.04)	.03	1.16 (0.26-5.20)	.84	1.13 (0.82-1.56)	.45	1.16 (0.92-1.47)	.21

Abbreviations: ARIC, Atherosclerosis Risk in Communities study; CHD, coronary heart disease; HR, hazard ratio; JHS, Jackson Heart Study; MESA, Multi-Ethnic Study of Atherosclerosis; REGARDS, Reasons for Geographic and Racial Differences in Stroke; SCT, sickle cell trait; WHI, Women’s Health Initiative.

^a^ Model 1 adjusted for age, sex, study region (REGARDS study only), and principal components analysis of global genetic ancestry; model 2 adjusted for components of model 1 and components of the Framingham CHD risk score (hypertension [defined as diagnosed or current use of antihypertensive medication] or a high systolic blood pressure), diabetes, cigarette smoking (current vs noncurrent except in the WHI and ARIC studies, in which the data were ever smokers vs never smokers), and serum total cholesterol and/or high-density lipoprotein cholesterol levels; model 3 adjusted for components of model 2 and income, educational level, serum high-sensitivity C-reactive protein level, and history of statin use (WHI analysis also adjusted for trial group among WHI participants); and model 4 adjusted for components of model 3 and glomerular filtration rate, estimated from the CKD-Epi equation.

^b^ For models 3 and 4 in the WHI study, the total sample size was 5744, with 473 individuals with SCT.

**Figure.**
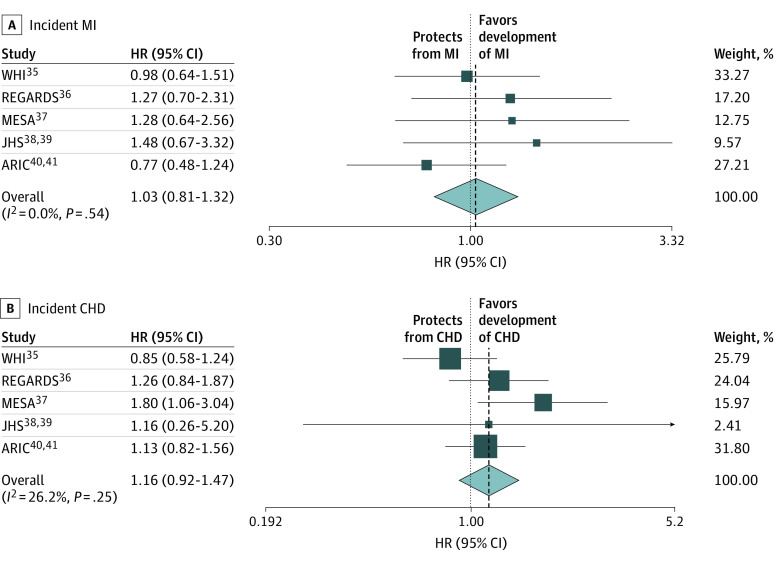
Random-Effects Meta-Analysis of the Fully Adjusted Cox Proportional Hazards Regression Model A, Association of sickle cell trait with incident myocardial infarction (MI). B, Association of sickle cell trait with incidence of any coronary heart disease (CHD). The follow-up periods were 1993 and 1998 to 2014 for the Women’s Health Initiative (WHI) study, 2003 to 2014 for the Reasons for Geographic and Racial Differences in Stroke (REGARDS) study, 2002 to 2016 for the Multi-Ethnic Study of Atherosclerosis (MESA), 2002 to 2015 for the Jackson Heart Study (JHS), and 1987 to 2016 for Atherosclerosis Risk in Communities (ARIC) study. Squares represent means, with horizontal lines representing 95% CIs. Diamonds represent the pooled means with the points of the diamonds representing the 95% CIs of the pooled means. The dotted vertical lines indicate the directionality of the overall effect. The sizes of the data markers indicate the individual weight for each study. HR indicates hazard ratio.

## Discussion

In this study, we sought to examine whether SCT was independently associated with an increased incidence of MI or CHD in African American individuals. The premise is based on studies showing that SCT was associated with putative factors (inflammation and thrombosis) associated with CHD in addition to CKD, which is associated with atherosclerotic CVD.^19,27,49,50^ In this analysis of 23 197 unrelated individuals from 5 different population-based cohort studies, our main finding was that SCT was not associated with the incidence of MI or CHD in both unadjusted and fully adjusted (accounting for factors associated with CVD) models.

Furthermore, in fully adjusted models, significant associations were not present between SCT and incident MI within each cohort or after meta-analysis of all 5 studies. In addition, SCT was not independently associated with increased risk of the composite CHD outcome. Although unadjusted meta-analyzed incidence rates for CHD were higher among SCT carriers than among non-SCT carriers, the fully adjusted meta-analyzed hazard ratio for the composite CHD outcome was not significant. Both SCT risk estimates for MI and the composite CHD outcome were not statistically significant in the meta-analyses.

An examination of the results in each cohort indicated that, among participants with SCT in the REGARDS study, the MESA, and the JHS, the risk of MI was slightly higher compared with that among participants without SCT, although the difference was not statistically significant. Also, the broad 95% CIs indicate a less-robust estimate of risk within each of these cohorts. However, estimates in the MESA cohort revealed that SCT was independently associated with a significantly higher risk of the composite CHD outcome. As was the case in the MI analysis, the risk of CHD was slightly higher among participants with SCT in the REGARDS study, JHS, and ARIC study cohorts than among those without SCT, but the difference was not statistically significant. The reasons for these differences in the association of SCT with CHD in the MESA and the other cohorts is not known but might be related to the higher proportion of male participants in the MESA cohort (46%) compared with the other cohorts (approximately 8% excluding the WHI study), differences in age, and/or the prevalence of other factors associated with CHD. This point is supported by 2 studies.^51,52^ One showed that SCT was associated with a higher risk of retinopathy among men, but not women, with diabetes,^51^ and the second showed that SCT was associated with coronary artery disease among men, but not women, with CKD.^52^ Despite having a higher proportion of men with SCT than men without SCT, the JHS cohort did not show a significant association between SCT and CHD, which could be owing to the small number of CHD events in this cohort. However, sex likely did not have a significant effect on our results because our analysis was adjusted for sex and we expect this adjustment to have addressed any differential effect of sex. Future studies could explore this factor because the cohorts analyzed in this study were not well powered to perform a sex-stratified analysis.

Our study is, to our knowledge, the largest to examine the association between SCT and incidence of MI and CHD and, as such, adds to the current literature. The findings may provide useful information leading to a closer examination and a more thorough workup for a patient with SCT who presents with CHD symptoms.

### Limitations

This study has limitations. We were unable to account for the modifying effect of other genetic factors, such as alpha-thalassemia, which is a modifier of the effect of the sickle β-globin variant,^53,54^ although this variant has not been associated with CHD. We were also unable to stratify our analysis by sex owing to the limited number of events within sex strata; thus, we could not confirm the sex differences described in an earlier study.^52^ Future studies with larger samples will be needed to properly address the question of whether SCT is associated with MI or CHD alone or whether the association varies by sex and other factors. In addition, we were not able to stratify our analysis by the different CHD components, for instance, by examining individuals who underwent procedures (coronary artery bypass grafting or percutaneous transluminal coronary angioplasty) for CHD.

## Conclusions

In this cohort study of 5 prospective studies, there was not a significant association between SCT and an increased risk of MI or CHD in African American individuals. Although prior studies showed that SCT is associated with sudden death,^55,56^ our findings suggest that these disorders may not be associated with SCT-related sudden death.

## Notes

Supplement. **eMethods.**
