# Supplementary material for: Association of Sickle Cell Trait With Incidence of Coronary Heart Disease Among African American Individuals
Source: JAMA Netw Open. 2021 Jan 5;4(1):e2030435. doi: 10.1001/jamanetworkopen.2020.30435 (PMC7786247; doi:10.1001/jamanetworkopen.2020.30435)
Supplement: Supplement. — eMethods. [file jamanetwopen-e2030435-s001.pdf]

Supplementary Online Content

Hyacinth HI, Franceschini N, Seals SR, et al. Association of sickle cell trait with incidence of coronary heart disease among African American individuals. *JAMA Netw Open.* 2021;4(1):e2030435. doi:10.1001/jamanetworkopen.2020.30435

eMethods.

This supplementary material has been provided by the authors to give readers additional information about their work.

© 2021 Hyacinth HI et al. *JAMA Network Open.*

eMethods.

Cohort Descriptions

Women's Health Initiative (WHI)

WHI is one of the largest prospective population-based cohort study investigating post-menopausal women's health in the United States (U.S). A total of 161,808 women aged 50–79 years old were recruited from 40 U.S. clinical centers between 1993 and 1998 to participate in the observational study (OS) and in clinical trials (CT): postmenopausal hormone replacement therapy (estrogen alone or estrogen plus progestin), a calcium and vitamin D supplement trial, and a dietary modification trial.[1] A diverse population including 26,045 (17%) women from minority groups were recruited from 1993-1998 at 40 clinical centers across the U.S. Recruitment was done through mass mailing to a list of age-eligible women obtained from voter registration, driver's license and Health Care Financing Administration or other insurance databases, with emphasis on recruitment of minorities and older women. Exclusions included participation in other randomized trials, predicted survival < 3 years, alcoholism, drug dependency, mental illness, and dementia. For the clinical trial, women were ineligible if they had a systolic BP > 200 mm Hg or diastolic BP > 105 mm Hg, a history of hypertriglyceridemia or endometrial cancer.

A total of 5,904 subjects from WHI (having the relevant phenotype and covariate data) were included in the current study. All 5,904 were directly genotyped for rs334 using direct Taqman®.

REason for Geographic And Racial Difference in Stroke (REGARDS)

REGARDS is a longitudinal community-based cohort study designed to investigate the factors associated with excess stroke mortality among blacks and residents of the "stroke belt" region of the United States.[2] The stroke belt encompasses the 8 Southern states of North Carolina, South Carolina, Georgia, Tennessee, Mississippi, Alabama, Louisiana, and Arkansas.[3] REGARDS

© 2021 Hyacinth HI et al. *JAMA Network Open.*

sampled a total of 30,239 community-dwelling adult individuals aged ≥45 years, who self-reported as Non-Hispanic Black or White. Sampling was started in January, 2003 and was completed by October, 2007. Potential enrollees where determined from a well characterized commercially available list from the GENESYS database/sampling system. The exclusion criteria included any self-reported medical conditions (e.g. cancer) that would prevent long-term participation, or being on a waiting list for a nursing home. The sample design was such that participants were balanced on race and sex, and across the stroke buckle and stroke belt, and the rest of the contiguous United States. The resulting sample was such that, 21% were from the buckle of the stroke belt, 35% from the rest of the stroke belt area (i.e. minus the buckle) and 44% from the other 40 contiguous states of the United States. Further, 42% of the total sample were Blacks, and 55% were women.

Initial study contact between participants and interviewers was via a mailed questionnaire. This was subsequently followed by an initial in-home visit where informed consent was obtained and blood samples collected for various laboratory measures. Participants or their proxies were contacted every six months to obtain medical history and identify hospitalizations, emergency department visits, overnight stays in nursing homes or rehabilitation centers, or death within the last 6 months. For those who reported an event such as CHD or MI, medical records were sought and events were adjudicated by an expert panel of trained adjudicators, based on pre-specified adjudication criteria (see below).

A total of 10,731 participants had direct Taqman® genotyping for rs334 of which 10,714 have the relevant phenotype and covariate data, and were included in this study.

© 2021 Hyacinth HI et al. *JAMA Network Open.*

Multi-Ethnic Study of Atherosclerosis (MESA)

The MESA study was designed to investigate the correlates of subclinical cardiovascular disease (CVD) progression in a longitudinal multi-ethnic cohort free of CVD at baseline. The details of the methods and design of the MESA study have been previously reported.[4] Participants were excluded if they had physician-diagnosed CVD prior to enrollment, including angina, myocardial infarction, heart failure, stroke or TIA, resuscitated cardiac arrest or a cardiovascular intervention (e.g., CABG, angioplasty, valve replacement, or pacemaker/defibrillator placement). Pre-specified recruitment plans identified four racial/ethnic groups (White European-American, African-American, Hispanic-American, and Chinese-American) for enrollment, with targeted oversampling of minority groups to enhance statistical power. Between July 2000 and August 2002, a total of 10,966 individuals were screened, and 6,814 individuals self-identified as white, African-American, Hispanic, or Chinese who were aged 45-84 years were deemed eligible and enrolled into the MESA study from 6 centers in the United States (Baltimore, MD; Chicago, IL; Forsyth County, NC; Los Angeles, CA; New York, NY; and St. Paul, MN). The institutional review board (IRB) at each participating institution approved the study, and all participants signed informed consents which also cover ancillary studies including this one.

For this study, participant characteristics were obtained from data collected at the enrollment visit from physical measures, standardized questionnaires, and laboratory tests. These data include demographic information (age, sex, race/ethnicity), medical history, medications, and alcohol and tobacco use. Resting blood pressure was determined by taking three measurements with the participant in the seated position. Systolic and diastolic blood pressures were recorded as the average value of the last two measurements from both the first and second study examinations. Since the onset of MESA, participants have returned for a total of 4 visits, in 2002–2004 (exam 2), 2004–2005 (exam 3) 2005–2007 (exam 4), and 2010-2012 (exam 5).

© 2021 Hyacinth HI et al. *JAMA Network Open.*

For this study, a total of 1,556 subjects (who had complete genotype, phenotype and covariate data) were included; out of which 152 were directly genotyped for rs334 via sequencing and the rest were imputed.

Jackson Heart Study (JHS)

The JHS is a single-site, prospective, population-based study designed to explore the environmental, behavioral, and genetic factors that influence the development of CVD among African Americans. A total of 5,301 women and men between the ages of 21 and 94 were recruited between 2000 and 2004 from a tri-county area of Mississippi: Hinds, Madison, and Rankin Counties. Participants were recruited from four sources, including (1) randomly sampled households from a commercial listing; (2) ARIC participants; (3) a structured volunteer sample that was designed to mirror the eligible population; and (4) a nested family cohort. Overviews of the JHS including the sampling and recruitment, sociocultural data, and laboratory methods have been described and published previously.[5-8] The institutional review boards of the following participating institutions approved the study: the University of Mississippi Medical Center, Jackson State University, and Tougaloo College. All participants provided written informed consent for the parent JHS and subsequent ancillary studies including this one. Unrelated participants were between 35 and 84 years old, and members of the family cohort were ≥ 21 years old when consent for genetic testing was obtained and blood was drawn for DNA extraction.

The baseline examination consisted of a home interview, self-administered questionnaires, and a clinic visit. Medications taken in the prior 2 weeks were brought to clinic and transcribed verbatim with subsequent coding by a pharmacist. After an overnight fast, anthropometric and seated blood pressure measurements were obtained and venipuncture/urine collection was performed in accordance with the National Committee for Clinical Laboratory Standards. Blood pressure was

© 2021 Hyacinth HI et al. *JAMA Network Open.*

measured by trained technicians using a Hawksley random zero manometer and determined by the arithmetic average of two readings taken 1 minute apart after a five-minute rest.[9]

A total of 2,175 subjects (having the relevant phenotype and covariate data) were included in the current study, of which 1,827 were directly genotyped for rs334 via sequencing, the rest were imputed.

Atherosclerosis Risk in Communities (ARIC) study

The Atherosclerosis Risk in Communities (ARIC) Study, sponsored by the National Heart, Lung and Blood Institute (NHLBI), is a prospective epidemiologic study conducted in four U.S. communities: Forsyth County, NC; Jackson, MS; the northwest suburbs of Minneapolis, MN; and Washington County, MD. A full description of the study has been previously published.[10] The ARIC study's goal is to investigate the etiology and natural history of atherosclerosis, the etiology of clinical atherosclerotic diseases, and variation in cardiovascular risk factors, medical care and disease by race, gender, location, and date.

A total of 15,792 participants (55% female and 27% African American) aged 45-64 years were recruited between 1987 and 1989 and received extensive examination; medical, social and demographic data were also collected. A total of 4,211 African Americans was enrolled in Jackson, Mississippi, and Forsyth County, North Carolina.[11] The baseline visit was conducted between 1987 and1989, the second visit in 1990-1992, the third visit in 1993-1995, the fourth visit in 1996-1998, the fifth visit in 2011-2013, the 6th visit in 2016-2017 and the 7th visit in 2018-2019. Participants were also contacted annually by telephone to maintain contact and to assess the health status of the cohort and participants, since 2012, telephone follow-up is now conducted semi-annually.

At each study visit, participants provided information on demographics and medical history, received blood pressure measurement and provided blood samples. Systolic and diastolic blood

© 2021 Hyacinth HI et al. *JAMA Network Open.*

pressures (BP) were recorded as an average of two of the last three measurements performed with the participant comfortably seated. Hypertension was defined as systolic BP≥140 mmHg, diastolic BP≥90 mmHg, or self-reported use of antihypertensive medication. Body mass index (kg/m^2) was calculated from weight and height measurements. Blood glucose was determined from blood samples collected during clinic visits. Diabetes was defined as fasting glucose≥126 mg/dl, non-fasting glucose≥200 mg/dl, self-reported physician diagnosis of diabetes, or self-reported use of oral hypoglycemic medication or insulin.[11]

The current study includes 2,848 African American subjects from ARIC who had provided consent for genetic studies and who had sufficient genetic material and data, all of whom were directly genotyped for rs334.

Exome Sequencing

Exome sequencing (N=2,052) was performed through the National Heart, Lung, and Blood Institute (NHLBI) Exome Sequencing Project (ESP) (CHS=90, MESA=146, JHS=367, WHI-567) and in the NHLBI Minority Health Genomics and Translational Research Bio-Repository Database (MH-GRID) (N=311 from JHS) as described in Fu et al.,[12] and in the National Institute of Diabetes and Digestive and Kidney Diseases (NIDDK) Type 2 Diabetes Genetic Exploration by Next-Generation Sequencing in Multi-Ethnic Samples (T2D-GENES) (N=571 from JHS).[13] In all three sequencing studies, the rs334 variant was covered at high depth (mean depth = 131(ESP), (MH-GRID), (T2D-GENES).

Genotyping and Imputation

Direct genotype data for the rs334 variant was obtained by custom genotyping from the REGARDS and ARIC study. Whole blood DNA was isolated from the buffy coat layer using the Gentra Puregene Blood Kit (Qiagen, Inc., Valencia, CA; www.qiagen.com). Carriers of HbS were identified from biallelic variation in the single nucleotide polymorphism, rs334. In addition, carriers

© 2021 Hyacinth HI et al. *JAMA Network Open.*

of HbC were identified from rs3393016. Genotyping was performed using functionally tested TaqMan® SNP Genotyping Assays in accordance with manufacturer protocols (Life Technologies, Grand Island, NY; www.lifetechnologies.com). The following custom primer and probe sequences were used to capture bi-allelic variation: rs334 (A/T) Forward-TCAAACAGACACCATGGTGCAT, Reverse-CCCCACAGGGCAGTAACG, VIC-CTGACTCCTGAGGAGAA-MGB, 6FAM-CTGACTCCTGTGGAGAA-MGB; and rs33930165 (A/G) Forward-AAACAGACACCATGGTGCATCT, Reverse-CCCCACAGGGCAGTAACG, VIC-CAGACTTCTCCTTAGGAGTC-MGB, 6FAM-ACTTCTCCTCAGGAGTC-MGB (designed on complement strand). PCR product in a 5.5 µL reaction volume was amplified utilizing 0.9 µM of each forward and reverse primer, 0.2 µM of each FAM and VIC sequence-specific probe, 3 ng DNA, and 1X TaqMan Universal PCR Master Mix containing AmpliTaq Gold DNA Polymerase and no AmpErase UNG. After an initial step of 10 min at 95ºC, the products were amplified using 50 cycles of 15 s at 92ºC and 1 min at 60ºC. Allele detection and genotype calling were performed using the ABI 7900HT and the Sequence Detection System software (Life Technologies, formerly Applied Biosystems). Quality control measures utilized a blind duplicate program in which 5% of samples were re-genotyped at random.

Imputation of the rs334 variant was carried out as described in Auer et al.[14] Briefly, 2,163 participants (1,692 AAs, 471 EAs) from ESP with Affymetrix 6.0 genome-wide genotyping data were selected to form an imputation reference panel. The imputation target panel consisted of 15,826 AAs from WHI, ARIC, MESA, CARDIA, and JHS, of which 6,664 had sufficient phenotype information to include in this analysis. Standard quality control measures were performed.[14] Both target and reference panels were pre-phased using BEAGLE.[15] The reference panel was then imputed into the target using minimac.[16] The imputation quality score Rsq (which is equivalent to the squared correlation between proximal imputed and genotyped SNP) was 0.86. Individuals with the rs334 genotype derived via imputation were coded as having 0, 1, or 2 risk alleles using

© 2021 Hyacinth HI et al. *JAMA Network Open.*

the most probable genotype. The imputation of genotype in these cohorts, have been done as part of multiple projects[17-20] and the similarity in description with this supplemental methods is due to the fact that the same set of data is being utilized for the current study. Difference in the number of participants between projects is due to phenotype and/or covariate missingness, which is purely random.

Using N=1,132 samples with both exome sequence and imputed genotype data from JHS, we validated our imputed genotypes (Table S1). The kappa correlation for sequenced and imputed values was 0.88 (95%CI 0.84 - 0.92).

Table S1: Cross tabulation of imputed genotypes versus genotypes via sequencing, for the rs334 variant in 1,132 samples from JHS.

**rs334 via imputation**	**rs334 via sequencing**			
	HbAA	HbAS	HBSS	Total
HbAA	1,018	6	0	1,024
HbAS	15	91	0	106
HbSS	0	1	1	2
Total	1,033	98	1	1,132

Definition of and ascertainment of Incident MI and CHD by cohort

© 2021 Hyacinth HI et al. *JAMA Network Open.*

WHI

The details of the method for ascertainment and adjudication of cardiovascular disease, specifically coronary heart disease (CHD) phenotypes among WHI participants has already been published.[21] But briefly, WHI participants were contacted every 6 months or 12 months for those in clinical trials or observational study arm respectively; for medical history updates with regards to incident cardiovascular and specifically coronary heart disease outcomes. Any report of occurrence of coronary heart disease outcome in a participant was then investigated and adjudicated by a panel of independent physician adjudicators. If the coronary disease outcome is a myocardial infarction (MI), only the first one is adjudicated. Note that an MI following an angina or any other non-MI CHD outcome also required adjudication.[21] MI and CHD events were defined based on established criteria using a combination medical history, serum cardiac enzyme and electrocardiographic findings.[22]

REGARDS

A full and detailed method for case ascertainment and adjudication has been published.[23] But in brief, the REGARDS – MI Study was the ancillary study component of REAGRDS under which the acute MI and CHD phenotype data for this study were generated. All acute MI and CHD events in REGARDS were ascertained and adjudicated by a team of experts, using published guidelines.[24,25] For MI, medical records were examined for the presence of signs and symptoms of ischemia, such as abnormal cardiac enzymes and electrocardiographic (ECG) changes. Classification of MI and CHD events were guided by the Minnesota code.[26,27] MI were classified as “definite MI” i.e. those with diagnostic levels of serum cardiac enzyme and ECG findings. Probable MI were those with elevated but non-diagnostic serum cardiac enzyme levels and a positive but non-diagnostic ECG finding or with diagnostic ECG findings but missing cardiac enzyme results. For the purpose of this study, Definite or probable MI were classified as MI.

© 2021 Hyacinth HI et al. *JAMA Network Open.*

Composite CHD phenotype included fatal and non-fatal MI, coronary revascularization procedures, and non-MI fatal or non-fatal CHD.

MESA

In addition to the standard MESA examinations, participants or next of kin were contacted by telephone every 9-12 months for information on interim changes in the medical status of participants. Copies of death certificates, and medical records of hospitalizations and selected outpatient cardiovascular diagnoses were obtained. Incident MI and/or CHD outcomes were adjudicated by an expert panel of trained physician reviewers. Cardiovascular outcomes were judged as either “Definite”, “Probable” or “No/Absent”. The MESA criteria for diagnosing MI was adapted from the Atherosclerosis Risk in Communities (ARIC) study and utilized a combination of clinical information such as chest pain, cardiac enzymes and ECG changes. The full details of cardiovascular event ascertainment and adjudication in MESA have already been published [4,28]

JHS

In addition to the standard JHS examinations, participants were contacted by telephone annually beginning in 2005 to obtain interim information about cardiovascular disease related hospitalizations. A full detail of the methodology for cardiovascular disease (CHD, Stroke and CHF) event ascertainment and adjudication has been published.[29] Briefly, participants were contacted annually by phone. The medical records of participants who reported a CHD event were then abstracted and sent, along with relevant laboratory results, to a team of independent experts who adjudicated the event. Incident CHD events were defined as those with 1) an eligible discharge code and 2) occurring after enrollment in the JHS and 3) in a participant with a valid JHS code identification number. CHD diagnosis were made using a combination of medical

© 2021 Hyacinth HI et al. *JAMA Network Open.*

history, serum cardiac enzyme levels and ECG findings. They are classified using the Minnesota code as “Definite”, “Probable” or “No/Absent”.

ARIC

In addition to the standard ARIC examinations, participants were contacted by telephone annually, and hospital surveillance in the ARIC communities was performed to obtain interim information about hospitalizations. A complete detail of the methodology for ascertainment of and adjudication of cardiovascular events has already been published.[30] Briefly, MI or CHD events were identified based on a review of the medical and death records by ARIC staff members. MI was diagnosed based on clinical symptoms, abnormal cardiac enzyme levels and ECG findings. A computer algorithm was used to assign electrocardiographic (ECG) diagnosis based on the Minnesota code. An independent expert physician reviews cases for which there is a disagreement between the discharge diagnosis and the computer assigned diagnosis. MI were classified as “Definite”, “Probable”, “Suspect” or “No/Absent”. Fatal CHD was classified on the basis of chest pain, prior history of CHD, and underlying cause of death.

In all cohorts, definite and probable MI were combined to define MI. For this study, we only analyzed incident MI or CHD. Participants with a prior history of MI or CHD were excluded from our analysis.

© 2021 Hyacinth HI et al. *JAMA Network Open.*

References

1. Borel MJ, Buchowski MS, Turner EA, Goldstein RE, Flakoll PJ. Protein turnover and energy expenditure increase during exogenous nutrient availability in sickle cell disease. *The American journal of clinical nutrition.* 1998;68(3):607-614.
2. Howard VJ, Cushman M, Pulley L, et al. The Reasons for Geographic and Racial Differences in Stroke Study: Objectives and Design. *Neuroepidemiology.* 2005;25(3):135-143.
3. Howard G, Anderson R, Johnson NJ, Sorlie P, Russell G, Howard VJ. Evaluation of Social Status as a Contributing Factor to the Stroke Belt Region of the United States. *Stroke; a journal of cerebral circulation.* 1997;28(5):936-940.
4. Bild DE, Bluemke DA, Burke GL, et al. Multi-Ethnic Study of Atherosclerosis: objectives and design. *Am J Epidemiol.* 2002;156(9):871-881.
5. Taylor HA, Jr. The Jackson Heart Study: an overview. *Ethnicity & disease.* 2005;15(4 Suppl 6):S6-1-3.
6. Carpenter MA, Crow R, Steffes M, et al. Laboratory, reading center, and coordinating center data management methods in the Jackson Heart Study. *The American journal of the medical sciences.* 2004;328(3):131-144.
7. Fuqua SR, Wyatt SB, Andrew ME, et al. Recruiting African-American research participation in the Jackson Heart Study: methods, response rates, and sample description. *Ethnicity & disease.* 2005;15(4 Suppl 6):S6-18-29.
8. Payne TJ, Wyatt SB, Mosley TH, et al. Sociocultural methods in the Jackson Heart Study: conceptual and descriptive overview. *Ethnicity & disease.* 2005;15(4 Suppl 6):S6-38-48.
9. Wyatt SB, Akylbekova EL, Wofford MR, et al. Prevalence, awareness, treatment, and control of hypertension in the Jackson Heart Study. *Hypertension.* 2008;51(3):650-656.

© 2021 Hyacinth HI et al. *JAMA Network Open.*

10. The Atherosclerosis Risk in Communities (ARIC) Study: design and objectives. The ARIC investigators. *American journal of epidemiology.* 1989;129(4):687-702.

11. Foster MC, Coresh J, Fornage M, et al. APOL1 variants associate with increased risk of CKD among African Americans. *Journal of the American Society of Nephrology : JASN.* 2013;24(9):1484-1491.

12. Fu W, O'Connor TD, Jun G, et al. Analysis of 6,515 exomes reveals the recent origin of most human protein-coding variants. *Nature.* 2013;493(7431):216-220.

13. Sigma Type 2 Diabetes Consortium, Williams AL, Jacobs SB, et al. Sequence variants in SLC16A11 are a common risk factor for type 2 diabetes in Mexico. *Nature.* 2014;506(7486):97-101.

14. Auer PL, Johnsen JM, Johnson AD, et al. Imputation of exome sequence variants into population-based samples and blood-cell-trait-associated loci in African Americans: NHLBI GO Exome Sequencing Project. *The American Journal of Human Genetics.* 2012;91(5):794-808.

15. Browning SR, Browning BL. Rapid and accurate haplotype phasing and missing-data inference for whole-genome association studies by use of localized haplotype clustering. *Am J Hum Genet.* 2007;81(5):1084-1097.

16. Howie B, Fuchsberger C, Stephens M, Marchini J, Abecasis GR. Fast and accurate genotype imputation in genome-wide association studies through pre-phasing. *Nat Genet.* 2012;44(8):955-959.

17. Naik RP, Derebail VK, Grams ME, et al. Association of sickle cell trait with chronic kidney disease and albuminuria in African Americans. *JAMA.* 2014;312(20):2115-2125.

18. Lacy ME, Wellenius GA, Sumner AE, et al. Association of Sickle Cell Trait With Hemoglobin A1c in African Americans. *Jama.* 2017;317(5):507-515.

© 2021 Hyacinth HI et al. *JAMA Network Open.*

19. Bello NA, Hyacinth HI, Roetker NS, et al. Sickle cell trait is not associated with an increased risk of heart failure or abnormalities of cardiac structure and function. *Blood.* 2017;129(6):799-801.

20. Naik RP, Wilson JG, Ekunwe L, et al. Elevated D-dimer levels in African Americans with sickle cell trait. *Blood.* 2016;127(18):2261-2263.

21. Curb JD, McTiernan A, Heckbert SR, et al. Outcomes ascertainment and adjudication methods in the Women's Health Initiative. *Ann Epidemiol.* 2003;13(9 Suppl):S122-128.

22. Ives DG, Fitzpatrick AL, Bild DE, et al. Surveillance and ascertainment of cardiovascular events. The Cardiovascular Health Study. *Ann Epidemiol.* 1995;5(4):278-285.

23. Safford MM, Brown TM, Muntner PM, et al. Association of race and sex with risk of incident acute coronary heart disease events. *JAMA.* 2012;308(17):1768-1774.

24. Luepker RV, Apple FS, Christenson RH, et al. Case definitions for acute coronary heart disease in epidemiology and clinical research studies: a statement from the AHA Council on Epidemiology and Prevention; AHA Statistics Committee; World Heart Federation Council on Epidemiology and Prevention; the European Society of Cardiology Working Group on Epidemiology and Prevention; Centers for Disease Control and Prevention; and the National Heart, Lung, and Blood Institute. *Circulation.* 2003;108(20):2543-2549.

25. Thygesen K, Alpert JS, White HD, et al. Universal definition of myocardial infarction. *Circulation.* 2007;116(22):2634-2653.

26. Prineas R, Blackburn H. *The Minnesota code manual of electrocardiographic findings: standards and procedures for measurement and classification. .* Boston, MA: John Wright-PSG Inc; 1982.

27. Prineas RJ, Crow RS, Zhang Z-M. *The Minnesota code manual of electrocardiographic findings.* 2nd ed. London, England: Springer-Verlag; 2010.

© 2021 Hyacinth HI et al. *JAMA Network Open.*

28. Bluemke DA, Kronmal RA, Lima JA, et al. The relationship of left ventricular mass and geometry to incident cardiovascular events: the MESA (Multi-Ethnic Study of Atherosclerosis) study. *J Am Coll Cardiol.* 2008;52(25):2148-2155.

29. Keku E, Rosamond W, Taylor HA, Jr., et al. Cardiovascular disease event classification in the Jackson Heart Study: methods and procedures. *Ethnicity & disease.* 2005;15(4 Suppl 6):S6-62-70.

30. Rosamond WD, Chambless LE, Heiss G, et al. Twenty-two-year trends in incidence of myocardial infarction, coronary heart disease mortality, and case fatality in 4 US communities, 1987-2008. *Circulation.* 2012;125(15):1848-1857.

© 2021 Hyacinth HI et al. *JAMA Network Open.*
